# Relationship of the Content of Systemic and Endobronchial Soluble Molecules of CD25, CD38, CD8, and HLA-I-CD8 and Lung Function Parameters in COPD Patients

**DOI:** 10.1155/2017/8216723

**Published:** 2017-08-07

**Authors:** Nailya Kubysheva, Larisa Postnikova, Svetlana Soodaeva, Viкtor Novikov, Tatyana Eliseeva, Ildar Batyrshin, Timur Li, Igor Klimanov, Alexander Chuchalin

**Affiliations:** ^1^Kazan Federal University, Kremlyovskaya St, 18, Kazan 420000, Russia; ^2^Nizhny Novgorod State Medical Academy, Minin and Pozharsky Square 10/1, Nizhny Novgorod 603005, Russia; ^3^Pulmonology Research Institute, 11-Parkovaya 32, Moscow 105077, Russia; ^4^I.M. Sechenov First Moscow State Medical University, Trubetskaya, 8- 2, Moscow, Russia; ^5^Lobachevsky State University of Nizhny Novgorod, Gagarina Avenue 23, Nizhny Novgorod 603950, Russia; ^6^Centro de Investigación en Computación, Instituto Politécnico Nacional (CIC-IPN), Av. Juan de Dios Bátiz, Esq. Miguel Othón de Mendizábal S/N, Gustavo A. Madero, 07738 Mexico City, Mexico; ^7^Central Clinical Hospital of RAS, Litovskiy Blvd. 1A, Moscow 117593, Russia

## Abstract

The definition of new markers of local and systemic inflammation of chronic obstructive pulmonary disease (COPD) is one of the priority directions in the study of pathogenesis and diagnostic methods improvement for this disease. We investigated 91 patients with COPD and 21 healthy nonsmokers. The levels of soluble CD25, CD38, CD8, and HLA-I-CD8 molecules in the blood serum and exhaled breath condensate (EBC) in moderate-to-severe COPD patients during exacerbation and stable phase were studied. An unidirectional change in the content of sCD25, sCD38, and sCD8 molecules with increasing severity of COPD was detected. The correlations between the parameters of lung function and sCD8, sCD25, and sHLA-I-CD8 levels in the blood serum and EBC were discovered in patients with severe COPD. The findings suggest a pathogenetic role of the investigated soluble molecules of the COPD development and allow considering the content of sCD8, sCD25, and sHLA-I-CD8 molecules as additional novel systemic and endobronchial markers of the progression of chronic inflammation of this disease.

## 1. Introduction

A steady system progression with local chronic inflammation and partially reversible airway obstruction is the cornerstone for the modern concept of the pathogenesis of COPD. Inflammatory process in COPD has the multiple-factor nature and represents a complex system of inflammatory cell interaction, cytokines, growth factors, and changes in the expression of membrane differentiation molecules [1–4].

It is known that proteins presented in the cell membrane of the immune system may have soluble homologues formed resulting in alternative splicing of prematrix RNA and/or proteolytic shedding from the cell surface [5–11]. Soluble forms of membrane molecules participate in immunological reactions at different stages of the immune response implementation. Violation of their equilibrium contents in biological environments leads to modulation of intercellular membrane interactions and, respectively, the immune response. For this reason, soluble forms of membrane proteins can be considered as endogenous immune-regulatory molecules integrated into the global immunological network. Information about a change direction in their concentration can have important predictive value for immune-mediated diseases of various genesis, including COPD.

Initiation and progressing of the inflammation in COPD are in many respects caused by an accumulation of the activated macrophages, neutrophils, and lymphocytes in a respiratory tract which play an essential role in the development of pathological processes in airways of patients [12, 13]. Activation of immune cells is accompanied by the raised expression on their surface of activation membrane proteins and soluble form emergence of their membrane analogues in biological fluids.

The soluble forms of CD25 (sCD25, sIL-2), CD38 (sCD38), and CD8 (sCD8) molecules are the activation cell markers having homologous receptors on their surface. These proteins are formed from an enzymatic cleavage of surface protein and possess pleiotropic functions. Ligand binding and prevention of its interaction with the membrane form of the antigen, as well as transsignal function, are one of the key mechanisms of immunomodulatory and regulatory action of soluble forms of membrane molecules [3–5].

The soluble form of the CD25 protein is capable of competing with an IL-2 membrane receptor and by the principle of feedback can limit hyperactivation of the immune system [14–17]. The sCD38 protein reduces CD38-mediated signal transduction, and by that blocks immune-regulatory functions of a membrane form of this protein [18].

Activated CD8^+^ cells play a leading role in the development of the pathogenetic mechanisms of COPD since the mediators produced by these cells have a destructive effect on the lung tissue [19, 20]. CD8 protein performs the adhesive and costimulatory molecule function of the T cell receptor and can exist in a soluble form. Since soluble CD8 molecules are derived structures of activated CD8^+^ cells, an elevated level of these molecules can serve as a marker for the activation of cytotoxic lymphocytes [21]. It is known that the activation of CD8^+^ cells occurs with the direct molecule participation of the major histocompatibility complex class I (HLA-I). From their interaction, soluble forms of CD8 and HLA-I are released into the extracellular environment [5, 21]. Binding to each other, these molecules may form protein nanostructures constructed of soluble CD8 protein and HLA-I–sHLA-I-CD8.

It should be noted that the data concerning the involvement of soluble differentiation membrane molecules in COPD pathogenesis are rare and contradictory [22–24], which emphasize the importance of further studying the role of these molecules in the development of inflammation in COPD.

The definition of new inflammatory markers at both systemic and local levels is one of the priority directions for studying the pathogenesis of COPD. Currently, the use of noninvasive inflammation biomarkers to control the effectiveness of therapy for obstructive lung diseases is actively being studied [24–26]. Therefore, content research of soluble differentiation molecules in the exhaled breath condensate in COPD patients could be relevant and promising.

The aims of this study were to evaluate the content of sCD25, sCD38, and sCD8 molecules and complexes of sHLA-I-CD8 in the blood serum and exhaled breath condensate and to determine the relationship concentration of these soluble molecules and the ventilation lung function in patients with moderate-to-severe COPD in different disease periods.

## 2. Materials and Methods

### 2.1. Study Population

The study involved 112 people, including 21 healthy nonsmokers (control group).

Patients with COPD (*n* = 91) were divided into four groups: patients with moderate COPD (GOLD II) during the exacerbation (*n* = 21) and in the stable phase (*n* = 23) and patients with severe COPD (GOLD III) during the exacerbation (*n* = 22) and in the stable phase (*n* = 25).

The written informed consent was obtained from all participants. The study was approved by the Ethics Committee of the Pulmonology Research Institute, Moscow, Russia.

COPD was defined and categorized according to the criteria of Global Initiative for Chronic Obstructive Lung Disease (GOLD) [27]. According to the definition of GOLD, an exacerbation of COPD is an acute event characterized by a worsening of the patient's respiratory symptoms that is beyond normal day-to-day variations and leads to a change in the medication [27]. The study included patients meeting the inclusion criteria: aged over 40, had smoking history of ≥10 pack-years, showed nonreversible postbronchodilator airflow obstruction (FEV_1_ < 80%, ≥30%; FEV_1_/FVC < 70%), clinical history focused on respiratory symptoms of COPD exacerbation, such as cough frequency and sputum characteristics, and administration of the modified Medical Research Council (mMRC) questionnaire for the assessment of dyspnoea severity.

The exclusion criteria are as follows: asthma and other allergic diseases, pneumonia, history of congestive heart failure, severe arterial hypertension, diabetes, and conditions requiring the long-term use of systemic corticosteroids. Pulmonary function study was carried out on a computer spirograph “SpiroLab III” (Italy) for the evaluation of the FEV_1_ and FEV_1_/FVC. The demographic and functional characteristics of the COPD patients are presented in Table 1.

### 2.2. Serum and Exhaled Breath Condensate Preparation

The blood samples were obtained in the morning on an empty stomach from the median cubital vein and then immediately centrifuged at 3000 rpm for 10 minutes. The serum was extracted and stored at −80°C.

The exhaled breath condensate was collected within 20 minutes after rinsing the mouth using system R-tube (USA).

### 2.3. Measurement of the Content of Soluble CD25, CD38, and CD8 Molecules and sHLA-I-CD8 Complex

The levels of soluble CD25, CD38, CD8, and sHLA-I-CD8 molecules in the serum and EBC were determined by enzyme-linked immunosorbent assay (ELISA) using an ELISA reader (Multiskan MS, Labsystems, Finland) at a wavelength of 405 nm. In determining the content of soluble CD25, CD38, and CD8 molecules, we used goat polyclonal antibodies against human leukocyte cell antigen and mouse monoclonal antibodies ICO-105, ICO-20, and ICO-31 against human CD25, CD38, and CD8 molecules conjugated with horseradish peroxidase as described in [28–31].

The content of soluble complexes of HLA-I-CD8 was determined using as a “first” antibodies for adsorption monoclonal antibodies ICO-53 against the HLA class I a chains, as immunoenzymic conjugate used monoclonal antibodies ICO-31 against CD8 molecules conjugated with horseradish peroxidase. The results were expressed in conventional units (U/ml).

### 2.4. Statistical Analysis

The data are presented as the mean ± SD. To determine the distribution normality, the Shapiro-Wilk test was used. Further analysis was performed by the ANOVA analysis and Student's *t*-test. To calculate the correlation coefficient (*r*), the Pearson correlation test was used. Statistical significance level was considered to be *p* < 0.05.

## 3. Results

**Figure 1:**
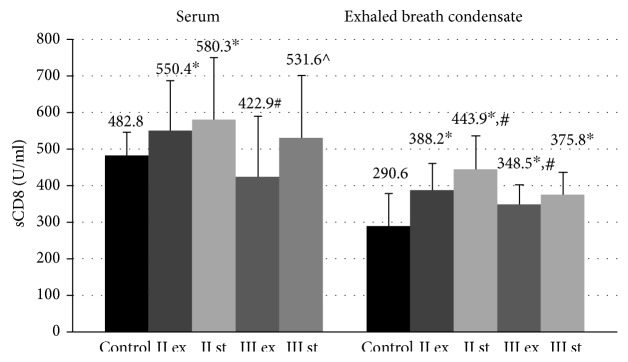
Concentration of sCD8 molecules in blood serum and exhaled breath condensate in COPD patients during the exacerbation and in the stable period. Data are presented as mean ± SD; control: healthy nonsmoking volunteers; II: moderate COPD; III: severe COPD; ex: exacerbation; st: stable phase; ^∗^*p* < 0.05 versus healthy nonsmokers; ^#^*p* < 0.05 versus patients with moderate COPD during the exacerbation; ^^^*p* < 0.05 versus patients with severe COPD during the exacerbation.

The serum levels of sCD8 molecules in GOLD II patients during the exacerbation (550.4 ± 135.5 U/ml, *p* = 0.041) and in the stable phase (580.3 ± 169.6 U/ml, *p* = 0.019) were statistically increased in comparison with nonsmoking volunteers (482.8 ± 63.1 U/ml) (Figure 1()). Within this group of patients, there were no significant differences in the serum content of sCD8 molecules between the exacerbation and the stable period.

In patients with GOLD III, the concentration of sCD8 molecules in the blood serum did not differ from the control values (*p* > 0.5). In severe COPD, the serum level of sCD8 during the exacerbation (422.9 ± 165.9 U/ml) was significantly lower in both the stable phase (531.6 ± 168.8 U/ml, *p* = 0.049) and in GOLD II patients with exacerbation (*p* = 0.016).

The concentration of these molecules in EBC in all examined patients was higher in comparison with nonsmoking volunteers (290.6 ± 87.9 U/ml, ANOVA *p* ≤ 0.001) (Figure 1). The downward trend in the topical sCD8 level was associated with the increase of COPD severity. The concentration of these proteins in EBC in COLD III patients during the exacerbation (348.5 ± 53.2 U/ml, *p* = 0.035) was lower than the corresponding values in the group of patients GOLD II in the same period of the disease (388.2 ± 71.16 U/ml, *p* = 0.035).

It was found that there was a significant difference in the topical concentration of sCD8 molecules between the exacerbation and the stable phase in GOLD II patients. The level of sCD8 proteins in EBC was lower during exacerbation in the group of moderate COPD (388.2 ± 71.16 U/ml) than in the stable period (443.9 ± 152.3, *p* = 0.031).

The positive correlation between the serum concentration of sCD8 and the parameter of lung function FEV_1_ (%) was found in patients with severe COPD (*r* = 0.5, *p* = 0.004) (Table 2).

The positive correlations between the topical concentration of these proteins and FEV_1_ (%) in moderate COPD (*r* = 0.55  *p* = 0.01) and in severe COPD patients (*r* = 0.43, *p* = 0.01) were detected. In addition, a positive association was found between the serum concentration of sCD8 molecules and FEV_1_ (%) in GOLD III patients (*r* = 0.5, *p* = 0.004) (Table 2).

**Figure 2:**
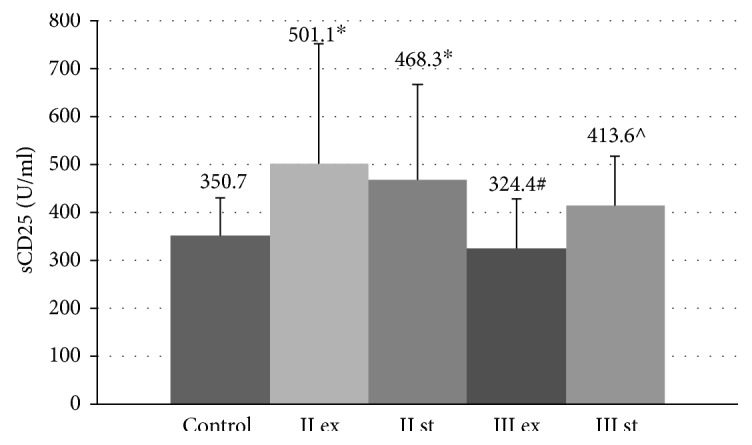
Serum concentration of sCD25 molecules in COPD patients during the exacerbation and in a stable period. Data are presented as mean ± SD; control: healthy nonsmoking volunteers; II: moderate COPD; III: severe COPD; ex: exacerbation; st: stable phase; ^∗^*p* < 0.05 versus healthy nonsmokers; ^#^*p* < 0.05 versus patients with moderate COPD during the exacerbation; ^^^*p* < 0.05 versus patients with exacerbation of severe COPD.

In our work, a progressive decrease in serum level of sCD25 in the blood serum of patients with COPD was linked to the growth of disease severity. The serum level of sCD25 molecules in all patients with moderate COPD was significantly higher than in a group of healthy nonsmoking volunteers (350.7 ± 79.4 U/ml, *p* < 0.01) (Figure 2). The content of these proteins in circulation in GOLD III patients did not differ from that in the control group (*p* > 0.05).

**Figure 3:**
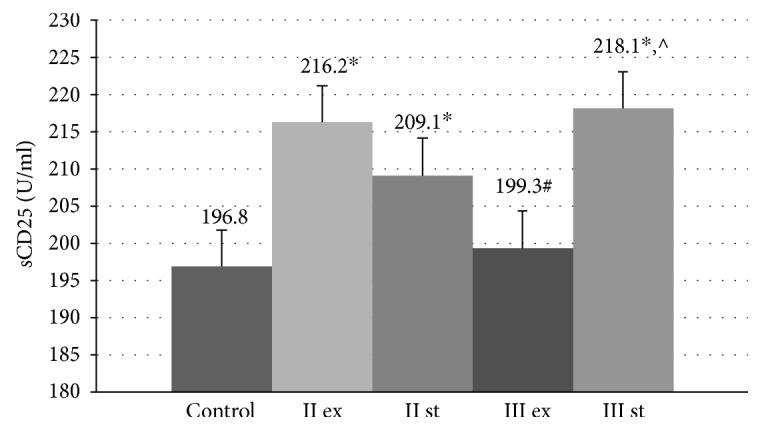
Concentration of sCD25 molecules in the exhaled breath condensate in COPD patients with exacerbation and in a stable period. Data are presented as mean ± SD; control: healthy nonsmoking volunteers; II: moderate COPD; III: severe COPD; ex: exacerbation; st: stable phase; ^∗^*p* < 0.05 versus healthy nonsmokers; ^#^*p* < 0.05 versus patients with moderate COPD during the exacerbation; ^^^*p* < 0.05 versus patients with severe COPD during the exacerbation.

The levels of sCD25 molecules in the EBC were statistically increased in GOLD II and in stable GOLD III compared to the control group (196.8 ± 16.1 U/ml, ANOVA *p* < 0.01) (Figure 3()).

Within the group of patients with moderate COPD, there were no significant differences in the content of sCD25 protein in the tested biological fluids between the exacerbation and stable period. At the same time, in patients with exacerbation of severe COPD, the concentrations of sCD25 molecules in the serum (324.4 ± 101.5 U/ml) and in EBC (199.3 ± 22.49 U/ml) were statistically lower than that in the group of stable patients (413.6 ± 102.2 U/ml *p* = 0.045 and 218.1 ± 25.14 U/ml *p* = 0.015, resp.).

The positive correlations between the serum and topical levels of sCD25 molecules and parameters of the lung function in patients with severe COPD were presented in Table 2.

**Figure 4:**
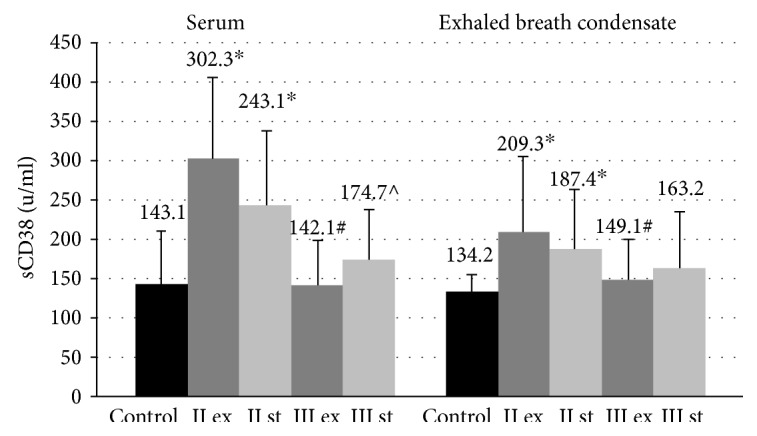
Concentration of sCD38 molecules in blood serum and exhaled breath condensate in COPD patients during the exacerbation and in a stable period. Data are presented as mean ± SD; control: healthy nonsmoking volunteers; II: moderate COPD; III: severe COPD; ex: exacerbation; st: stable phase; ^∗^*p* < 0.05 versus healthy nonsmokers; ^#^*p* < 0.05 versus patients with moderate COPD during the exacerbation; ^^^*p* < 0.05 versus patients with severe COPD during the exacerbation.

The levels of sCD38 proteins in GOLD II patients in the blood serum and EBC exceeded control values (143.4 ± 67.4 U/ml and 134.2 ± 21.5 U/ml, resp., *p* < 0.05) (Figure 4). The exacerbation of GOLD III was characterized by a decrease in the concentration of soluble CD38 molecules in the investigated biological fluids compared to the patients with COLD II in the same period.

**Figure 5:**
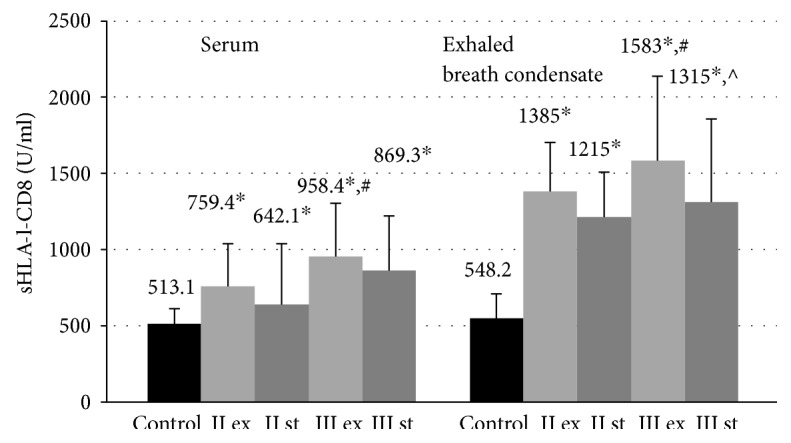
Concentration of sHLA-I-CD8 molecules in blood serum and exhaled breath condensate in COPD patients during exacerbation and in a stable period. Data are presented as mean ± SD; control: healthy nonsmoking volunteers; II: moderate COPD; III: severe COPD; ex: exacerbation; st: stable phase; ^∗^*p* < 0.05 versus healthy nonsmokers; ^#^*p* < 0.05 versus patients with moderate COPD during the exacerbation; ^^^*p* < 0.05 versus patients with severe COPD during the exacerbation.

sHLA-I-CD8 concentrations in the blood serum and EBC in all COPD patients exceed the corresponding values of healthy nonsmoking volunteers (513.1 ± 101.3 U/ml and 548.2 ± 161.6 U/ml, resp., ANOVA *p* < 0.01) (Figure 5()). It was found that the tendency towards growth in the content of these complexes in the serum and EBC was associated with the progression of COPD severity.

In patients with exacerbation of GOLD III, the sHLA-I-CD8 concentrations in the serum (958.4 ± 346.8 U/ml) and EBC (1583 ± 390.8 U/ml) were considerably higher than that in the group of patients COLD II in the same period of the disease (759.4 ± 279.1 U/ml and 1385 ± 220.8 U/ml, resp., *p* < 0.05). Within the group of patients GOLD II and GOLD III, significant differences in the content of sHLA-I-CD8 proteins in the analyzed biological fluids between the exacerbation and in the stable phase have not been revealed.

The negative correlation between the levels of sHLA-I-CD8 molecules in biological fluids and lung function parameters such as FEV_1_ (%) and FEV_1_/FVC (%) was established in patients with severe COPD (Table 2).

The high positive correlations between serum concentrations of sCD8, sCD25, and sCD38 have been detected in all examined COPD patients (Table 3). Moreover, the significant positive correlation between the serum concentration of complex sHLA-I-CD8 and the levels of sCD8 molecules (*r* = 0.49  *p* = 0.01) was established (Table 3).

The study revealed the positive association between the levels of sCD8 and the sCD25 content in the exhaled breath condensate (*r* = 0.33  *p* = 0.03) (Table 4).

## 4. Discussion

In our research, we found the unidirectional changes of serum concentration of sCD25, sCD38, and sCD8 dependent on disease severity in patients with COPD. The revealed positive correlations between the studied molecule content in the blood serum in COPD patients also emphasize the identity changes in the level of the soluble proteins under consideration, associated with the progression of the inflammatory process in COPD.

In GOLD II patients, the serum levels of sCD25, sCD38, and sCD8 molecules in the exacerbation and in the stable phase exceeded the biomarker values of healthy nonvolunteers. The concentrations of circulating sCD8, sCD25, and sCD38 proteins were significantly lower in severe COPD patients during all periods of the disease in comparison with the group of GOLD II patients and did not differ from the control group. In the exhaled breath condensate, the content of sCD25, sCD38, and sCD8 proteins decreased in patients with severe bronchial obstructive disorders only during the exacerbation compared to the moderate COPD in the same period.

Since sCD25, sCD38, and sCD8 are produced by activated mononuclear cells, their content in the biological fluids is considered as an indirect marker of immune system cell activation [15, 16]. Therefore, the increased serum levels of the studied molecules may indirectly reflect the increase in the functional activity of mononuclear cells both in the circulation and in the respiratory tract. Perhaps, the reduction in the content of sCD25, sCD38, and sCD8 proteins in patients with the severe disease to the reference level is due to the progressive oppression of the producer cell activity of these soluble molecules under expressed inflammation and hypoxemia conditions.

Functionally, soluble differentiation molecules are pleiotropic proteins. The function of sCD25 is in neutralizing IL-2-dependent immune responses by binding to circulating IL-2 [14, 15, 18]. It is likely that an elevated level of sCD25 in the mechanism of negative feedback limits the excessive activation of the immune system in GOLD II patients. At the same time, blocking of IL-2 receptors, for example, on the surface of natural killer (NK) cells, may lead to a suppression of the cytotoxic function of NK cells. Given that the main function of natural killers is in antiviral defense, perhaps, an increase in the level of sCD25 is one of the causes of an immune response infringement concerning infectious agents that can induce intensification and progression of inflammation in COPD. The rise in the content of sCD25 molecules in moderate COPD may further lead to suppression of immune responses and consequently reduce the content of these soluble molecules in severe disease. Probably, simultaneous decrease of sCD25 levels in the blood and EBC in patients with severe COPD initiates the formation of the immunological dysfunction and pathological events, leading not only to lung tissue damage but also to systemic inflammation progression.

The function of sCD38 molecules is also targeted to block the immune-regulatory activity of membrane protein form [32–34]. The soluble and membrane form of the CD38 molecule participates in the migration of leukocytes from circulation through the capillary wall to various organs, including the lungs and bronchi [32, 35]. The high content of sCD38 in patients with moderate COPD could be the result of a restriction mechanism aimed at inhibiting the excessive migration of effector cells into the inflammatory site. In this case, an increase in the content of these soluble molecules is an unfavorable factor from the point of view of normal resolution of inflammation, since the possibility of effector cells to realize their functions in the phlogogenic center is blocked. Consequently, there can be violations in mechanisms of the inflammatory process return development underlying the chronicity cornerstone and worsening the course of COPD.

It is possible that a decrease in sCD38 level in the biological fluids against a background of bronchial obstruction growth is due to a decrease in the membrane form expression of a similar protein and, accordingly, the limitation of shedding in the course of inflammation progression.

The action of soluble forms of CD8 molecules aims at cytotoxic cell deactivation by the feedback principle due to interaction with ligands of its membrane homologues [21]. In this case, the increase in the level of sCD8 in moderate COPD can affect a violation of an immune response in the implementation of anti-infectious protection. It is known that the mediators released by activated CD8^+^ cell damage bronchopulmonary tissue, inducing apoptosis of epithelial cells and alveolar macrophages [2, 3]. It is possible to assume that the increased content of the sCD8 molecules, suppressing the activity of cytotoxic cells, limits the destructive mechanisms of inflammation in COPD. In contrast, a decrease in the serum and endobronchial concentration of sCD8 proteins in patients with severe airway obstruction can induce an enhancement of apoptotic processes. This hypothesis is confirmed by the results of our previous study, which demonstrated the increase of apoptosis markers in the circulation and airways in patients with severe COPD [24].

Established positive associations between endobronchial and serum levels of sCD25 and sCD8 molecules and parameters of lung function in patients with severe COPD may indicate the involvement of these soluble molecules in the progression of bronchial patency disorders in expressive inflammation. Probably, the decrease in the concentrations of soluble sCD25 and sCD8 proteins in the blood serum and EBC are unfavorable prognostic factors in the development of COPD. In addition, exacerbation of severe COPD was characterized by a significant decrease in the level of studied proteins in the serum compared with a stable period. Thus, changes in the content of sCD25 and sCD8 can be considered as markers for the steady activation and progression of inflammation of COPD.

Soluble forms of membrane molecules are capable of interacting with ligands not only on the cell surface but also to form soluble complexes constructed from “receptor-ligand” pairs in the extracellular space [5]. For sCD8, such partner is a soluble molecule of histocompatibility class I. As a result of their interaction, such protein nanostructures as sHLA-I:CD8 are formed [21].

In our study, systemic and topical levels of sHLA-I-CD8 complex in studied patients with COPD were higher than values of the control group. Significant differences in the concentration of these molecules in the studied biological fluids in the stable period and exacerbation within each group of patients were not detected. Thus, the dynamics of sHLA-I-CD8 content did not depend on the COPD stage. At the same time, the exacerbation in patients with severe COPD was characterized by the highest level of these associative complexes in comparison with the group of patients GOLD II in the same period.

The presence of a negative statistically significant correlation between the serum and topical content of sHLA-I-CD8 and lung function parameters in patients with severe COPD indicates the involvement of these soluble complexes in violation of airway obstruction. This allows us to consider changes in the complex concentration as an indicator of the systemic and topical inflammation activity.

We had found a positive correlation between serum levels of sCD8 and sHLA-I-CD8 complexes. Thus, the reduction of sCD8 content with disease growth severity is possibly due to the binding of soluble CD8 proteins with sHLA-I molecules and the formation of sHLA-I-CD8 complexes. Functional capabilities of HLA-I to CD8 in the genesis of pathological states have not been fully determined.

It is obvious that the structural-functional condition of HLA-I-CD8 complex has an impact on their immunomodulatory properties. In the interaction of the “receptor-ligand” in the extracellular space, the active binding sites of these proteins are mutually blocked; therefore, there is a neutralization of action of these molecules. Thus, the molecules sCD8 and sHLA-I, being in the complexes, are not able to interact with their membrane receptors.

Individually, sHLA-I and sCD8 limit the activation of cytotoxic cells. In this case, the presence of sHLA-I-CD8 complexes in biological fluids can be considered as one of the mechanisms limiting the suppressor functions of these molecules with respect to cytotoxic cells. The increased formation of soluble HLA-I-CD8 molecule can lead to the partial removal of cytotoxic cell activity blocking and, consequently, to the enhancement of production of mediators responsible for the development of apoptosis processes. We assume that high serum and topical levels of HLA-I-CD8 promote the enhancement of lung tissue damage in patients with severe COPD.

An inflammation is dual in nature, and it can appear both as a protective-adaptive or pathological response. It is possible that the functions of the studied soluble molecules in conditions of chronic inflammation in COPD have the same dual character. Anyway, our reasoning requires further study and evaluation concerning the action of the presented soluble forms of differentiation molecules in the pathogenetic mechanisms of COPD development.

## 5. Conclusion

Summarizing the obtained results, we can conclude that studied soluble differentiation molecules make a significant contribution to the development of the pathogenetic mechanisms of COPD. The resulting correlations between the lung function parameters and the content of sCD8, sCD25, and sHLA-I-CD8 in patients with severe COPD make it possible to consider the concentration of these molecules as additional prognostic systemic and endobronchial marker of the progression of chronic inflammation in COPD.

The reduction of sCD8 and sCD25 levels in the blood serum and EBC can reflect an unfavorable prognosis of the further disease course and serve as a quantitative indicator of the exacerbation risk development in COPD patients with severe airway obstruction.

## Figures and Tables

**Table 1:** Characteristics of patients with exacerbation of COPD and healthy nonsmokers included in the study.

		COPD	
	Healthy nonsmokers	Moderate	Severe
Subjects (*n*)	21	44	47
Age (years)	53.1 ± 7.8	56.1 ± 4.6	63.2 ± 4.3
Smoking pack-years	0	37.5 ± 5.8	39.2 ± 4.2
FEV1 % pred	105.6 ± 3.8	62.8 ± 4.6	40.5 ± 3.9
FEV_1_/FVC%	108.1 ± 3.7	61.6 ± 4.4	49.1 ± 7.9

Data were presented as mean ± SD. COPD: chronic obstructive pulmonary disease; pack-years: number of cigarettes packs per day multiplied by the number of smoking years; FEV_1_: forced expiratory volume in one second; % pred: % predicted; FVC: forced vital capacity.

**Table 2:** Correlations between the concentrations of sCD8, sCD25, sCD38, and sHLA-I-CD8 molecules in biological fluids and the lung function parameters in COPD patients.

	GOLD II		GOLD III	
	FEV_1_ (%)	FEV_1_/FVC (%)	FEV_1_ (%)	FEV_1_/FVC (%)
sCD8 (serum)	*r* = 0.36 *p* = 0.08	*r* = 0.19 *p* = 0.32	*r* = 0.5 *p* = 0.004	*r* = 0.24 *p* = 0.17
sCD8 (EBC)	*r* = 0.55 *p* = 0.01	*r* = 0.1 *p* = 0.4	*r* = 0.43 *p* = 0.01	*r* = 0.25 *p* = 0.18
sCD25 (serum)	*r* = 0.2 *p* = 0.3	*r* = −0.11 *p* = 0.53	*r* = 0.46 *p* = 0.041	*r* = 0.33 *p* = 0.04
sCD25 (EBC)	*r* = 0.22 *p* = 0.26	*r* = 0.17 *p* = 0.43	*r* = 0.44 *p* = 0.01	*r* = 0.43 *p* = 0.02
sCD38 (serum)	*r* = 0.21 *p* = 0.23	*r* = 0.18 *p* = 0.36	*r* = 0.25 *p* = 0.18	*r* = 0.23 *p* = 0.21
sCD38 (EBC)	*r* = −0.12 *p* = 0.46	*r* = 0.1 *p* = 0.46	*r* = 0.3 *p* = 0.16	*r* = 0.24 *p* = 0.17
sHLA-I-CD8 (serum)	*r* = 0.08 *p* = 0.67	*r* = 0.24 *p* = 0.21	*r* = −0.36 *p* = 0.032	*r* = −0.35 *p* = 0.04
sHLA-I-CD8 (EBC)	*r* = 0.23 *p* = 0.1	*r* = −.04 *p* = 0.87	*r* = −0.46 *p* = 0.02	*r* = −0.43 *p* = 0.03

*r*: correlation coefficient; EBC: exhaled breath condensate; FEV_1_: forced expiratory volume in 1 second; FVC: forced vital capacity.

**Table 3:** Correlations between sCD25, sCD38, sCD8, and sHLA-I-CD8 levels in blood serum in COPD patients.

	sCD38	sCD25	sHLA-I-CD8
sCD25	—	—	*r* = 0.19 *p* = 0.32
sCD38	—	*r* = 0.78 *p* = 0.001	*r* = 0.3 *p* = 02
sCD8	*r* = 0.76 *p* ≤ 0.001	*r* = 0.57 *p* = 0.001	*r* = 0.49 *p* = 0.005

*r*: correlation coefficient.

**Table 4:** Correlations between sCD25, sCD38, sCD8, and sHLA-I-CD8 levels in exhaled breath condensate in COPD patients.

	sCD38	sCD25	sHLA-I-CD8
sCD25			*r* = 0.1 *p* = 0.8
sCD38		*r* = −0.16 *p* = 0.8	*r* = 0.3 *p* = 0.2
sCD8	*r* = −0.25 *p* = 0.35	*r* = 0.33 *p* = 0.03	*r* = 0.13 *p* = 0.53

*r*: correlation coefficient.

## References

[1] Chung K. F., Adcock I. M. (2008). Multifaceted mechanisms in COPD: inflammation, immunity, and tissue repair and destruction. *European Respiratory Journal*.

[2] Barnes P. J. (2008). Immunology of asthma and chronic obstructive pulmonary disease. *Nature Reviews Immunology*.

[3] Rovina N., Koutsoukou A., Koulouris N. G. (2013). Inflammation and immune response in COPD: where do we stand?. *Mediators of Inflammation*.

[4] Barnes P. J., Chowdhury B., Kharitonov S. A. (2006). Pulmonary biomarkers in chronic obstructive pulmonary disease. *American Journal of Respiratory and Critical Care Medicine*.

[5] Novikov V. V., Evsegneeva I. V., Karaulov A. V., Baryshnikov A. J. (2005). Soluble forms of membrane antigens of immune system cells in social infections. *Russian Journal Biotherapy*.

[6] Blasczyk R., Westhoff U., Grosse-Wilde H. (1993). Soluble CD4, CD8, and HLA molecules in commercial immunoglobulin preparations. *The Lancet*.

[7] Dobbe L. M., Stam N. J., Neefjes J. J., Giphart M. J. (1988). Biochemical complexity of serum HLA class I molecules. *Immunogenetics*.

[8] Zavazava N. (1998). Soluble HLA class I molecules: biological significance and clinical implications. *Molecular Medicine Today*.

[9] Meager A., Bird C., Mire-Sluis A. (1996). Assays for measuring soluble cellular adhesion molecules and soluble cytokine receptors. *Journal of Immunological Methods*.

[10] Rubin L. A., Nelson D. L. (1990). The soluble interleukin-2 receptor: biology, function, and clinical application. *Annals of Internal Medicine*.

[11] Levine S. J. (2004). Mechanisms of soluble cytokine receptor generation. *The Journal of Immunology*.

[12] Barnes P. J., Cosio M. G., Siafakas N. M. (2006). Cells and mediators of chronic obstructive pulmonary disease. *Management of Chronic Obstructive Pulmonary Disease*.

[13] Barnes P. J. (2009). The cytokine network in chronic obstructive pulmonary disease. *American Journal of Respiratory Cell and Molecular Biology*.

[14] Heaney M. L., Golde D. W. (1996). Soluble cytokine receptors. *Blood*.

[15] Ho A. D., Maruyama M., Maghazachi A., Mason J. R., Gluck S., Corringham R. E. (1994). Soluble CD4, soluble CD8, soluble CD25, lymphopoetic recovery and endogenous cytokines after high dose chemotherapy and blood stem cell transplantation. *Blood*.

[16] Grimaldi J. C., Balasubramanian S., Kabra N. H. (1995). CD38-mediated ribosylation of proteins. *Journal of Immunology*.

[17] Limas C. J., Goldenberg I. F., Limas C. (1995). Soluble interleukin-2 receptor levels in patients with dilated cardiomyopathy correlation with disease severity and cardiac autoantibodies. *Circulation*.

[18] Mallone R., Ferrua S., Morra M. (1998). Characterization of a CD38-like 78-kDa soluble protein released from B cell lines derived from patients with X-linked agammaglobulinemia. *The Journal of Clinical Investigation*.

[19] Kemeny D. M., Vyas M., Vukmanovic-Stejic M. J., Thomas A., Noble L. (1999). Loh. CD8 (+) T cell subsets and chronic obstructive pulmonary disease. *American Journal of Respiratory and Critical Care Medicine*.

[20] Saetta M., Baraldo S., Corbino L. (1999). CD8+ve cells in the lungs of smokers with chronic obstructive pulmonary disease. *American Journal of Respiratory and Critical Care Medicine*.

[21] Morgan C. L., Price C. P., Cohen S. B., Madrigal J. A., Newman D. J. (1999). Soluble CD8 stabilizes the HLA class I molecule by promoting β_2_M exchange: analysis in real-time. *Human Immunology*.

[22] Takabatake N., Nakamura H., Inoue S. (2000). Circulating levels of soluble Fas ligand and soluble Fas in patients with chronic obstructive pulmonary disease. *Respiratory Medicine*.

[23] Yasuda N., Gotoh K., Minatoguchi S. (1998). An increase of soluble Fas, an inhibitor of apoptosis, associated with progression of COPD. *Respiratory Medicine*.

[24] Kubysheva N., Soodaeva S., Postnikova L., Novikov V., Maksimova A., Chuchalin A. (2014). Associations between indicators of nitrosative stress and levels of soluble HLA-I, CD95 molecules in patients with COPD. *COPD: Journal of Chronic Obstructive Pulmonary Disease*.

[25] Konstantinidi E. M., Lappas A. S., Tzortzi A. S., Behrakis P. K. (2015). Exhaled breath condensate: technical and diagnostic aspects. *The Scientific World Journal*.

[26] Eliseeva T. I., Kulpina Y. S., Soodaeva S. K., Kubysheva N. I. (2010). Content of the nitrogen oxide metabolites in a condensate of exhaling air in children with a bronchial asthma control different level. *Sovremennye Tehnologii v Medicine*.

[27] Global Initiative for Chronic Obstructive Lung Disease (GOLD) (2011). Global strategy for the diagnosis, management, and prevention of chronic obstructive pulmonary disease. *Vancouver (WA): Global Initiative for Chronic Obstructive Lung Disease (GOLD)*.

[28] Lebedev M. J., Krizhanova M. A., Vilkov S. A. (2003). Peripheral blood lymphocytes immunophenotype and serum concentration of soluble HLA class I in burns patients. *Burns*.

[29] Novikov V. V., Mamaeva M. E., Aliasova A. V. (2015). The serum level of total and oligomeric fractions of soluble molecules cd38 under malignant tumors of cervix and uterine body. *Klinicheskaia Laboratornaia Diagnostika*.

[30] Novikov V. V., Egorova N. I., Kurnikov G. Y., Evsegneeva I. V., Baryshnikov A. Y., Karaulov A. V. (2007). Serum levels of soluble HLA and IL-2R molecules in patients with urogenital chlamydia infection. *In Immune-Mediated Diseases Springer New York*.

[31] Lebedev M. J., Egorova N. I., Sholkina M. N., Vilkov S. A., Baryshnikov A. J., Novikov V. V. (2004). Serum levels of different forms of soluble CD38 antigen in burned patients. *Burns*.

[32] Pfister M., Ogilvie A., da Silva K., Grahnert A., Guse A. H., Hauschildt S. (2001). NAD degradation and regulation of CD38 expression by human monocytes/macrophages. *European Journal of Biochemistry*.

[33] Malavasi F., Funaro A., Roggero S., Horenstein A., Calosso L., Mehta K. (1994). Human CD38: a glycoprotein in search of a function. *Immunology Today*.

[34] Funaro A., Horenstein A. L., Calosso L. (1996). Identification and characterization of an active soluble form of human CD38 in normal and pathological fluids. *International Immunology*.

[35] Alessio M., Roggero S., Funaro A. (1990). CD38 molecule: structural and biochemical analysis on human T lymphocytes, thymocytes, and plasma cells. *Journal of Immunology*.

